# Scientific literature on infectious diseases affecting livestock animals, longitudinal worldwide bibliometric analysis

**DOI:** 10.1186/s13567-015-0280-2

**Published:** 2016-03-14

**Authors:** Christian Ducrot, Marjolaine Gautret, Thierry Pineau, André Jestin

**Affiliations:** UR0346 Epidémiologie animale, INRA, 63122 Saint Genès Champanelle, France; Département santé animale, INRA, 78352 Jouy-en-Josas, France; Département santé animale, INRA, 31027 Toulouse, France; Direction Scientifique, Anses, 94701 Maisons-Alfort, France

## Abstract

The objectives of this bibliometric analysis of the scientific literature were to describe the research subjects and the international collaborations in the field of research on infectious diseases in livestock animals including fishes and honeybees. It was based on articles published worldwide from 2006 through 2013. The source of data was the Web of Science, Core collection^®^ and only papers fully written in English were considered. Queries were built that combined 130 descriptors related to animal species and 1213 descriptors related to diseases and pathogens. To refine and assess the accuracy of the extracted database, supplementary filters were applied to discard non-specific terms and neighbouring topics, and numerous tests were carried out on samples. For pathogens, annotation was done using a thematic terminology established to link each disease with its corresponding pathogen, which was in turn classified according to its family. A total of 62 754 articles were published in this field during this 8-year period. The average annual growth rate of the number of papers was 5%. This represents the reference data to which we compared the average annual growth rate of articles produced in each of the sub-categories that we defined. Thirty-seven percent of the papers were dedicated to ruminant diseases. Poultry, pigs and fishes were covered by respectively 21, 13 and 14% of the total. Thirty-seven percent of papers concerned bacteria, 33% viruses, 19% parasites, 2% prions, the remaining being multi-pathogens. Research on virology, especially on pigs and poultry, is increasing faster than the average. There also is increasing interest in monogastric species, fish and bees. The average annual growth rate for Asia was 10%, which is high compared to 3% for Europe and 2% for the Americas, indicating that Asia is currently playing a leading role in this field. There is a well established network of international collaborations. For 75% of the papers, the co-authors were from the same country, for 10%, they were from different countries on the same continent, and for 15%, they were from different continents. The annual growth rate of papers representing international collaborations generally is increasing more quickly than the overall average.

## Introduction

Animal diseases can cause serious social, economic and environmental damage and in some cases they can also threaten human health. Depending on the impacts of global change, many threats arising out of the livestock industry and many zoonotic infectious diseases display global-scale features. In addition, the cost of developing and maintaining research infrastructure on infectious diseases is increasing due to the greater sophistication of technical research approaches as well as the progressive upgrade of safety regulations for bio-contained research facilities. Meanwhile, funds available to cover research running costs are becoming scarce. For these reasons, a global strategic alliance for the coordination of research on the major infectious diseases of animals was launched in 2011. The aim was to improve the coordination of research activities on the major infectious diseases of livestock and zoonoses in order to speed up the transfer of improved control methods to the farming sector.

This was planned to be achieved through the establishment of an international strategic forum of R&D programme owners/managers and international organisations called Star-Idaz [1]. Funded by the European Union, the purpose of Star-Idaz was to share information, improve collaboration on research activities and work towards common research agendas and coordinated research funding on the major animal diseases affecting livestock production and/or human health.

In order to achieve improvements in that domain, it was necessary to draw an overall picture of worldwide research activities in the field of emerging and major infectious diseases of livestock, including fish and honeybees, and those livestock infections that pose a risk for human health. Diseases of wildlife animals were also considered when these species were identified as reservoirs of infection involving emerging and major infectious diseases of humans or livestock animals.

Apart from mapping the research institutions and infrastructures through a web survey in partner countries of the project [2], an important task was to make a comprehensive map of research activities in the targeted domains through a thorough analysis of the scientific literature. Jean de Rycke produced an initial report when the Star-Idaz project was launched [3] that focused on partner countries and explored the scientific literature produced from 2006 through June 2010. The analysis of research outputs in specific research fields was carried out at two different levels, countries and research institutions, with a focus on co-publication networks.

The aim of the current bibliometric analysis is slightly different. It is based on an updated publications database which includes articles published worldwide in English from 2006 through 2013 (an 8-year period) in international peer-reviewed journals. The analysis aims to describe the research subjects and international collaborations in the field of infectious diseases of livestock animals, including fishes and honeybees, at a country scale, and their trends over time.

## Materials and methods

### Scope

The scope of the study was to analyze the scientific papers in international journals which were fully published in English in the field of infectious diseases in livestock animals. Studies on bees and fish were included. Studies on wild animals were considered relevant as long as they represented a direct or indirect source of disease transmission to livestock or humans. A methodological challenge was to retrieve and eliminate from this literature the papers obtained in murine models that were not related to infectious diseases in livestock animals, and to keep those that referred to mice as models for livestock animals.

The database on animal infectious diseases was designed and implemented using the Web of Science Core collection^®^ as our bibliographic resource. In accordance with the scope of the study, queries were built that combined 130 descriptors related to animal species (all species that can be raised by humans including some wild animal species, as well as wild animal species that can be a source of major pathogens for farm animals or humans), 1213 descriptors related to diseases and infectious agents, the period of publication (2006–2013) and the type of document (primary articles or reviews). The list of diseases and infectious agents, as well as their synonyms, were defined with experts on infectious diseases, and through a comprehensive search of all names of diseases and agents referred to in the CAB abstracts. To refine and assess the accuracy of the extracted database, additional filters were designed and applied to remove non-specific terms and neighbouring topics such as human diseases, food safety concerns and other wild animal diseases. Numerous validation tests also were carried out on article samples of intermediate size. A more detailed explanation of queries and filters will be found in Boissy et al. [4].

### Database

The preliminary database was established with selected papers relevant to the scope of the study which were accessible from the Web of Science Core collection^®^. It included describing parameters such as author names, title, journal, year, bibliographic references, summary, keywords. Additional fields for species groups, pathogens and geographical regions were developed and documented for each publication. For pathogens, annotation was done using a thematic terminology established to link each disease with its corresponding pathogen, which was in turn classified according to its family.

The term “other”, associated with bacteria, viruses or parasites was used for papers with agents unassigned in families. “Miscellaneus” was used when the taxonomy families were not specified in the article. “Multipathogen” was used when pathogens of different families were referred to in the article, and “multispecies” when several species of farm animals were mentioned in the article.

### Analysis

The study used an integer count to measure the contribution of partners to a research activity. If an article had multiple authors from different countries or continents, the article was affiliated with each of those countries or continents, with one paper credited to each of the countries or continents involved. Thus, any given article can account for multiples countries and continents.

Bibliometrics refers to a statistical procedure used to describe or quantify according to pre-set objectives. Quantification allowed us to establish overall trends and to highlight connections or correlations which would have remained hidden within large amounts of data. When a combination of analysis parameters led to data sets that were too small, the analyses were dicontinued and referred as “*ns*” (non-significant) in the tables. On a given topic, 400 publications was chosen as the threshold to sustain reliable studies for the period 2006–2013.

The average annual growth rate was calculated to highlight increases and decreases in the temporal evolution of the number of publications. Growth rate shows the evolution between the first and last year of the period; average annual growth rate is the arithmetic mean of the growth rate per year.

Average annual growth rate: $AAGR_{t, t + n} = \left( {\sqrt[n]{{\frac{{x_{t + n} }}{{x_{t} }} - 1}}} \right) \times 100$ where $x$ is the number of publications; $n$ is the number of years; and ($t, \;t + n$) is the period.

## Results

### Volume of scientific literature

**Figure 1:**
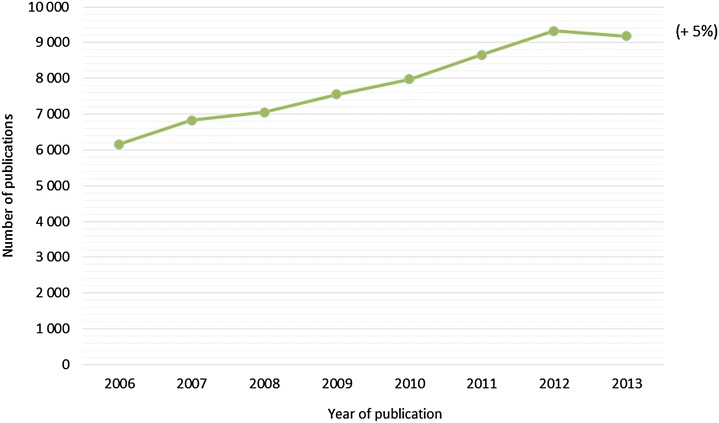
**Evolution of the number of publications for the period 2006–2013 (in brackets, average annual growth rate, for the period 2006–2013).**

A total of 62 754 articles on infectious diseases in livestock animals were published wordwide between 2006 and 2013. The database is available online [5]. The number of papers published on infectious diseases in livestock animals has increased progressively from 6000 per year in 2006 to over 9000 in 2013 (Figure 1). The slight drop in the curve observed in 2013 is due to the fact that not all of the papers published in 2013 were yet incorporated in the Web of Science Core collection^®^ at the time the data set was completed. Estimated on the basis of the increase trend observed from 2006 through 2012, the real number of papers published in 2013 might be close to 10 000.

Over the period analysed, the average annual growth rate of the number of papers on infectious diseases in livestock animals was 5% (7% over the 2006–2012 period).

This is important to keep in mind when analysing the scientific production in different thematic fields. As a preliminary example, the average annual growth rate of scientific production for bacteria was 4%, which means that this research domain is increasing at a rate slightly lower than the overall trend (5%).

**Figure 2:**
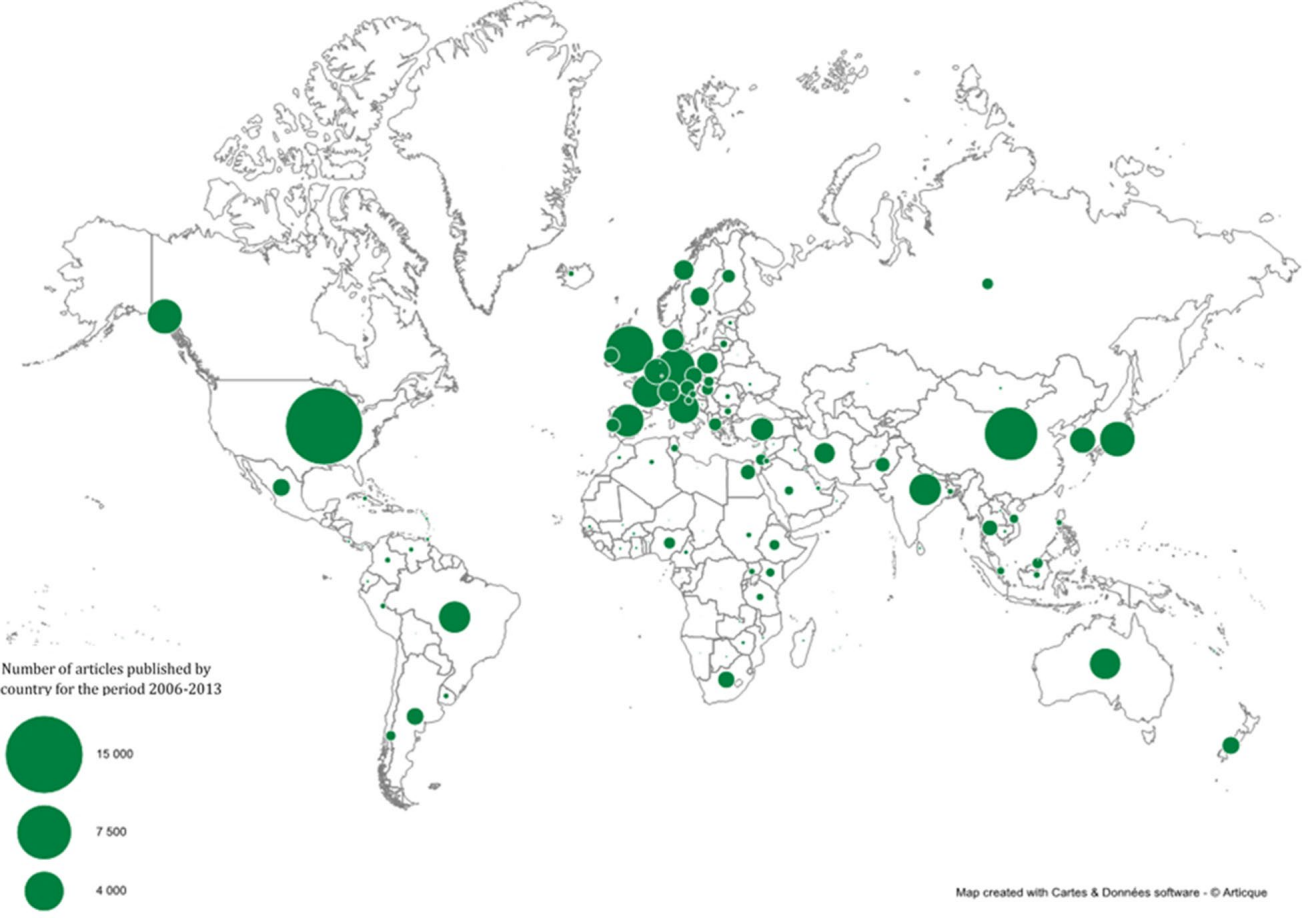
**Number of articles published by country for the period 2006–2013.**

The geographical distribution of the number of papers published during the 8-year period is presented at the country level in Figure 2.

**Figure 3:**
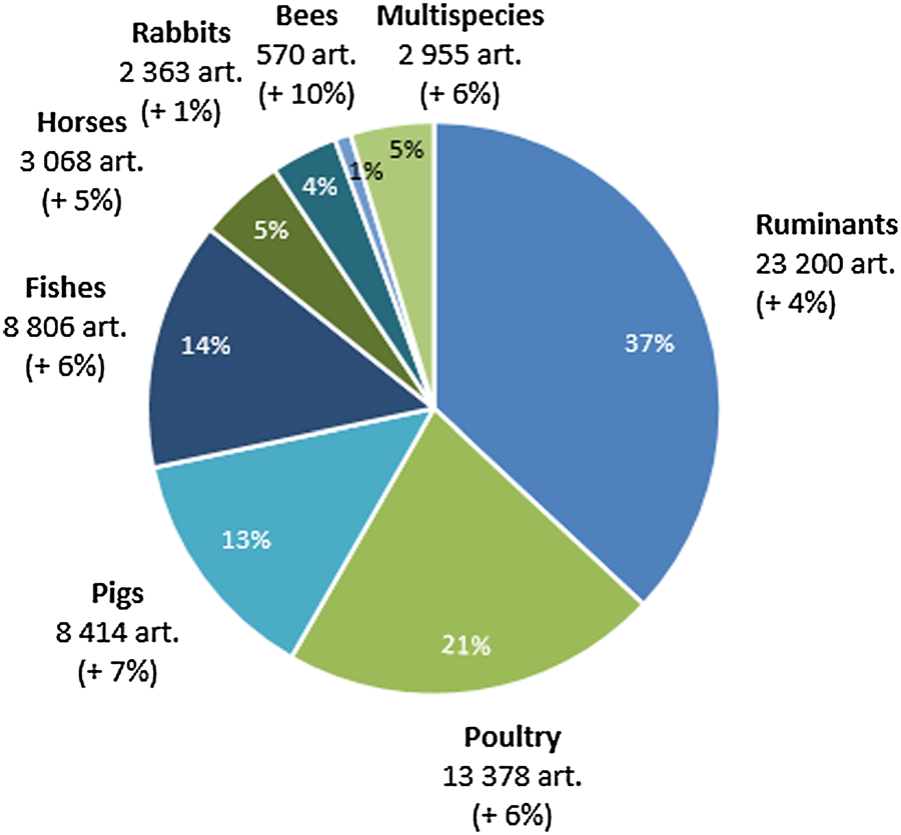
**Distribution of the number of articles by each animal species group for the period 2006–2013 (inner edge of the diagram, share of articles; in brackets, significant average annual growth rate, for the period 2006–2013).**

More than one-third (37%) of the total number of articles were dedicated to ruminant diseases, representing a total of 23 200 articles (Figure 3). Poultry, pigs and fishes represented respectively 21, 13 and 14% of the total number of articles. Articles dedicated to horse and rabbit diseases represented respectively 5 and 4%. Bees represented a minor group (1%), but the average annual growth rate was the highest (10%) compared to 5–7% for horses, fishes, pigs and poultry. The average annual growth rate was 4% for ruminants and 1% for rabbits.

### Research themes

#### Types of pathogens

**Figure 4:**
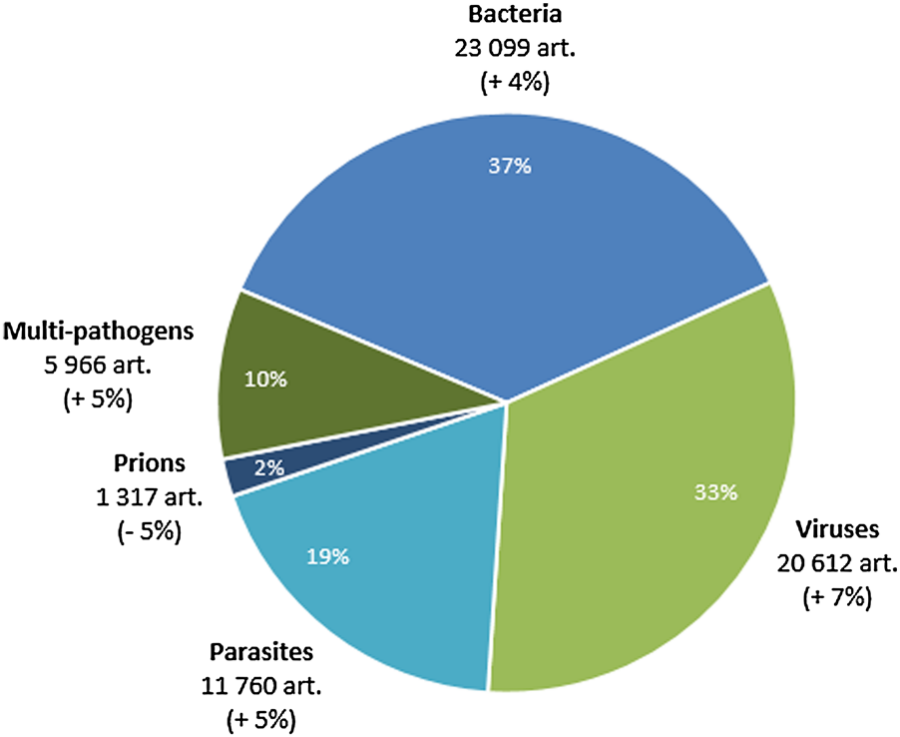
**Distribution of the number of articles by each pathogen group for the period 2006–2013 (inner edge of the diagram, share of articles; in brackets, significant average annual growth rate, for the period 2006–2013).**

Articles on bacteria, viruses and parasites represent respectively 37, 33 and 19% of the total number of articles for the 2006–2013 period (Figure 4), while corresponding respectively to 23 099, 20 612 and 11 760 articles. Articles dedicated to prions represent 2% of the global production, with 1317 articles, and the average annual growth rate is decreasing (−5%).

**Figure 5:**
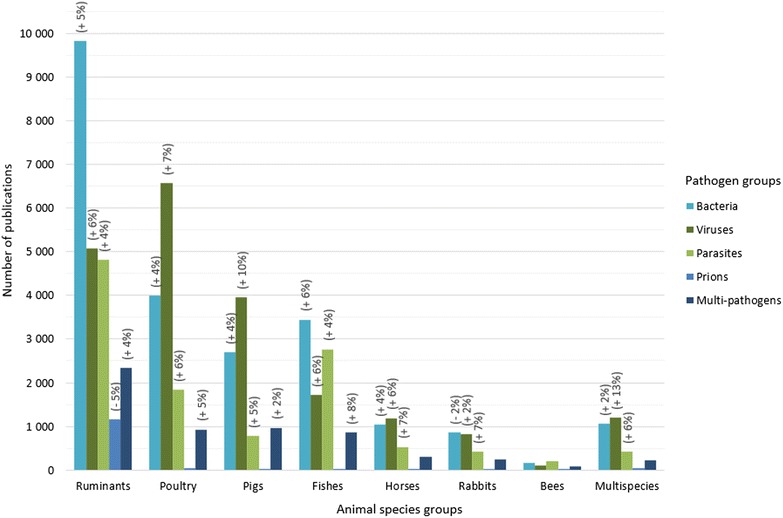
**Distribution of the number of articles by pathogen and animal species group for the period 2006–2013 (in brackets, significant average annual growth rate, for the period 2006–2013).**

Of the 23 200 publications on ruminants (Figure 5), 42% are related to ruminant diseases dealing with bacteria, and 21–22% to viruses and parasites. Average annual growth rates are in the same range (4–6%). In contrast, of the 13 378 publications on poultry, 49% of these articles are related to viral diseases, 30% to bacterial and 14% to parasitic diseases. Like ruminants, the average annual growth rates are comparable (4–7%). Pig analyses (8414 publications) display trends comparable to those of poultry; 47% of the articles are related to viruses, 32% to bacteria, and 9% to parasites. The average annual growth rates of reports on viral porcine diseases is increasing sharply (+10%), whereas for bacteria and parasites the rates are 4 and 5%, respectively.

The situation is different for fishes. Of the 8806 publications on fishes, bacteria and parasites are major groups, representing respectively 39 and 31% of the articles. Articles dedicated to fish viruses represent 19% of the total. Average annual growth rates are equivalent (4–6%). Horses have a different profile. Of the 3068 articles, papers dealing with viruses and bacteria represent respectively 39 and 34%. Articles dedicated to horse parasites represent 17%. Average annual growth rates are equivalent (4–7%). The profile for rabbits is similar to horses. Of the 2363 articles, publications dealing with viruses and bacteria represent respectively 34 and 37%. Articles dedicated to rabbit parasites represent 18%. Average annual growth rates are decreasing for bacteria (−2%) and increasing for viruses (2%) and parasites (7%). For bees, of the 570 publications, articles dealing with parasites and bacteria represent respectively 36 and 30%, articles dedicated to bee viruses represent 19%.

#### Families of pathogens

**Figure 6:**
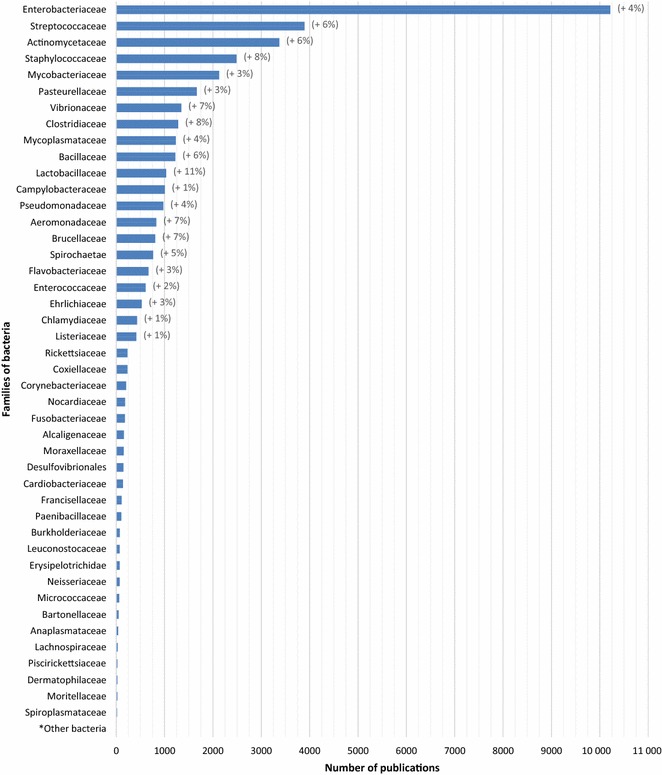
Distribution of the number of articles by each family of bacteria for the period 2006–2013 (in brackets, significant average annual growth rate, for the period 2006–2013; *unassigned families).

**Figure 7:**
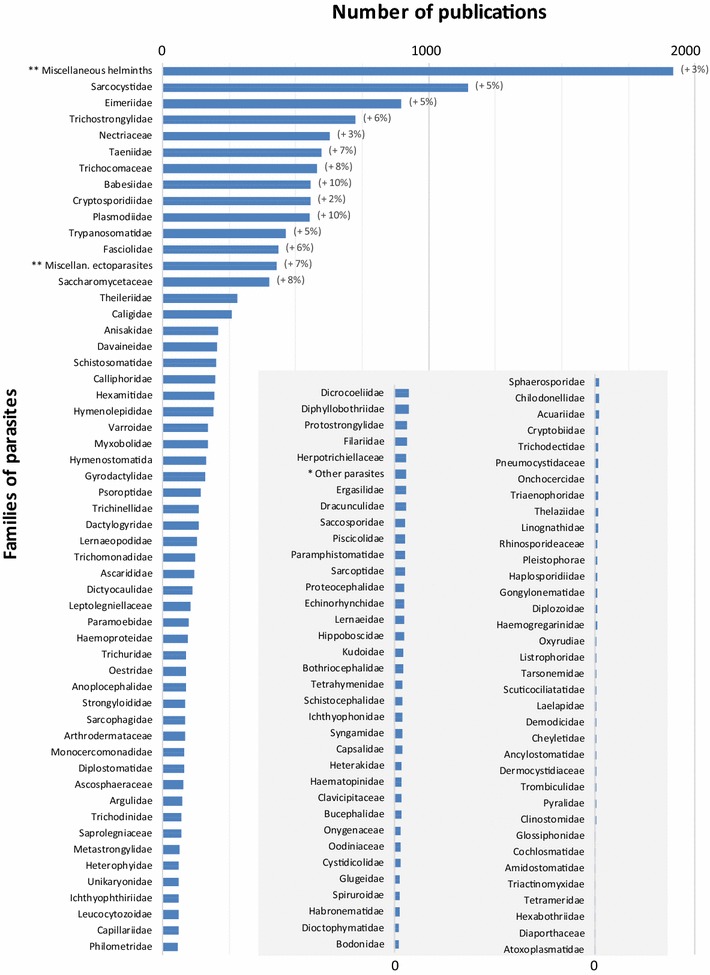
Distribution of the number of articles by each family of parasites for the period 2006–2013 (in brackets, significant average annual growth rate, for the period 2006–2013; *unassigned familied; **taxanomy families not specified in article).

**Figure 8:**
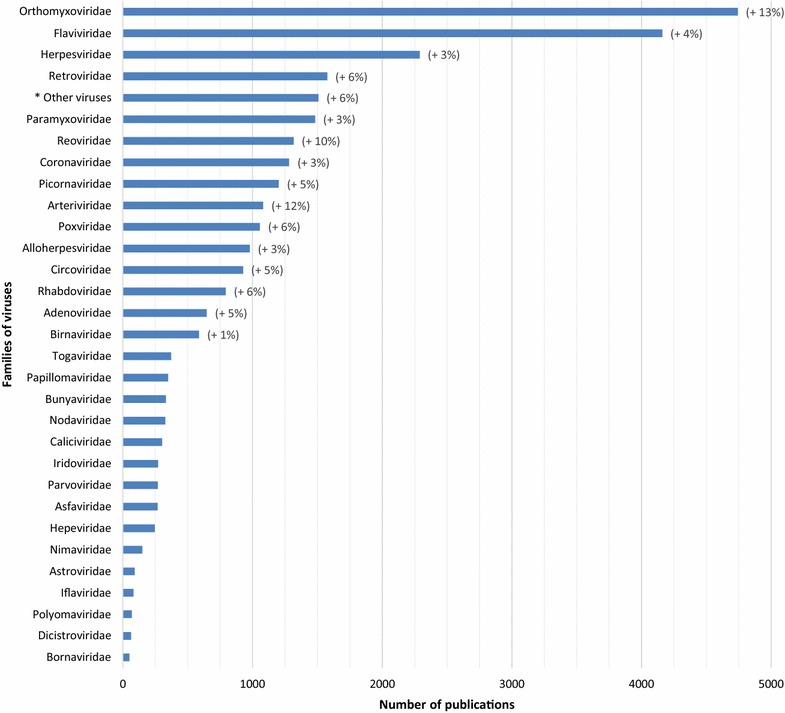
Distribution of the number of articles by each family of viruses for the period 2006–2013 (in brackets, significant average annual growth rate, for the period 2006–2013; *unassigned families).

The distribution of the number of papers published worldwide on the different families of pathogens is presented in Figures 6, 7 and 8 at the world level, respectively for bacteria, parasites and viruses. Detailed figures by continent are available in the report [6].

##### Bacteria

Enterobacteriaceae is the family that received the most coverage during the study period with 10 217 publications, representing 36% of the total amount (Figure 6). Streptococcaeae and Actinomycetaceae families came in second, representing respectively 14 and 12% of publications, followed by a third group which includes the Staphylococcaceae, Mycobacteriaceae and Pasteurellaceae families.

During this 8-year period, the trend was influenced by an 8% average annual growth rate of publications related to the Staphylococcaceae family, and a 7% average annual growth rate related to the Brucellaceae family. Mycobacteriaceae and Campylobacteraceae families continue to be areas of interest for scientific communities, but to a lesser extent as the average annual growth rates were respectively 3 and 1%, which are lower than the average (5%). The Lactobacillaceae family is studied extensively and its high average annual growth rate is related to publications dedicated to probiotics.

##### Parasites

Parasite families are not specified in several articles related to parasites. Instead, they are merged into a special group, the miscellaneous helminths, which were covered in 1919 articles, or 14% of the total (Figure 7). The second most represented group consists of the Sarcocystidae, Eimeriidae and Trichostrongylidae families, representing respectively 8, 6 and 5% of the total number of articles. During this 8-year period, the trend was dominated by a high average annual growth rate for Babesiidae (10%), Plasmodidae (10%) and Taeniidae (7%). In contrast, a low average annual growth rate was identified for the Cryptosporidiidae family (2%).

##### Viruses

The Orthomyxoviridae and Flaviviridae families received considerable attention over the entire 2006–2013 period (Figure 8). More than 4000 articles were published on each of these two families, representing respectively 19 and 16% of the total number of publications on viruses. The Herpesviridae, Retroviridae, Paramyxoviridae families form a second group, representing respectively 9, 6 and 6% of the publications on viruses. A third group includes the Reoviridae, Coronaviridae and Picornaviridae families. During the 8-year study period, the trend was dominated by a high average annual growth rate for the Orthomyxoviridae (13%), Arteriviridae (12%) and Reoviridae (10%) families. In contrast, a low average annual growth rate was identified for three viral families: Herpesviridae (3%), Paramyxoviridae (3%) and Birnaviridae (1%).

### Collaborations

#### International co-authorship

**Figure 9:**
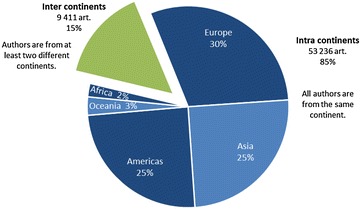
**Distribution of the number of articles by author’s home continent for the period 2006–2013 (inner edge of the diagram, share of articles).** Continents are defined by the United Nations Statistics Division.

The articles are attributed to continents according to authorship data (Figure 9). For 9411 articles (15%), the co-authors were from at least two different continents. Two groups emerge out of the intra-continent category. In the first group, Europe, the Americas and Asia produced 18 843, 15 519 and 15 686 articles, respectively representing 30, 25 and 25% of the total. In the second group, Oceania and Africa produced 1840 and 1348 articles, representing 3% and 2%. The average annual growth rates are 10 and 7% for Asia and Africa. The rates for Europe, 3%, and the Americas, 2%, are lower.

**Figure 10:**
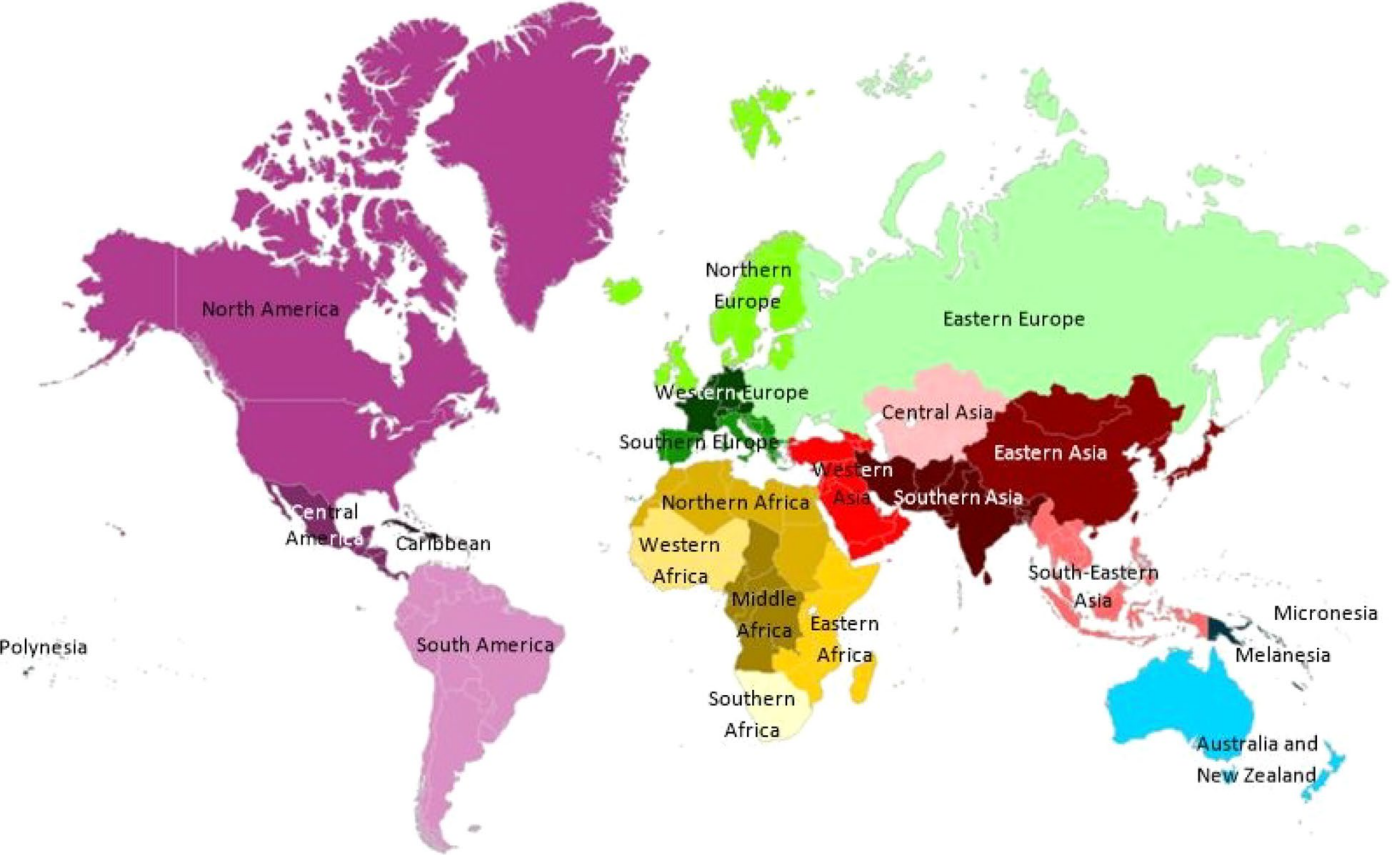
**Description of the geographical regions defined by the United Nations Statistics Division [** 5 **].**

The articles can be divided into three categories based on authorship. For 76% of the articles (47 487), all of the co-authors were from the same country (identified in Figure 10 as “national partnership”); for 9% of the articles (5749), the co-authors were from different countries of the same continent (identified in the figure as “intra continental partnership”), and for 15% of the articles (9411), the co-authors were from at least two different continents (identified in the figure as “extra continental partnership”).

**Figure 11:**
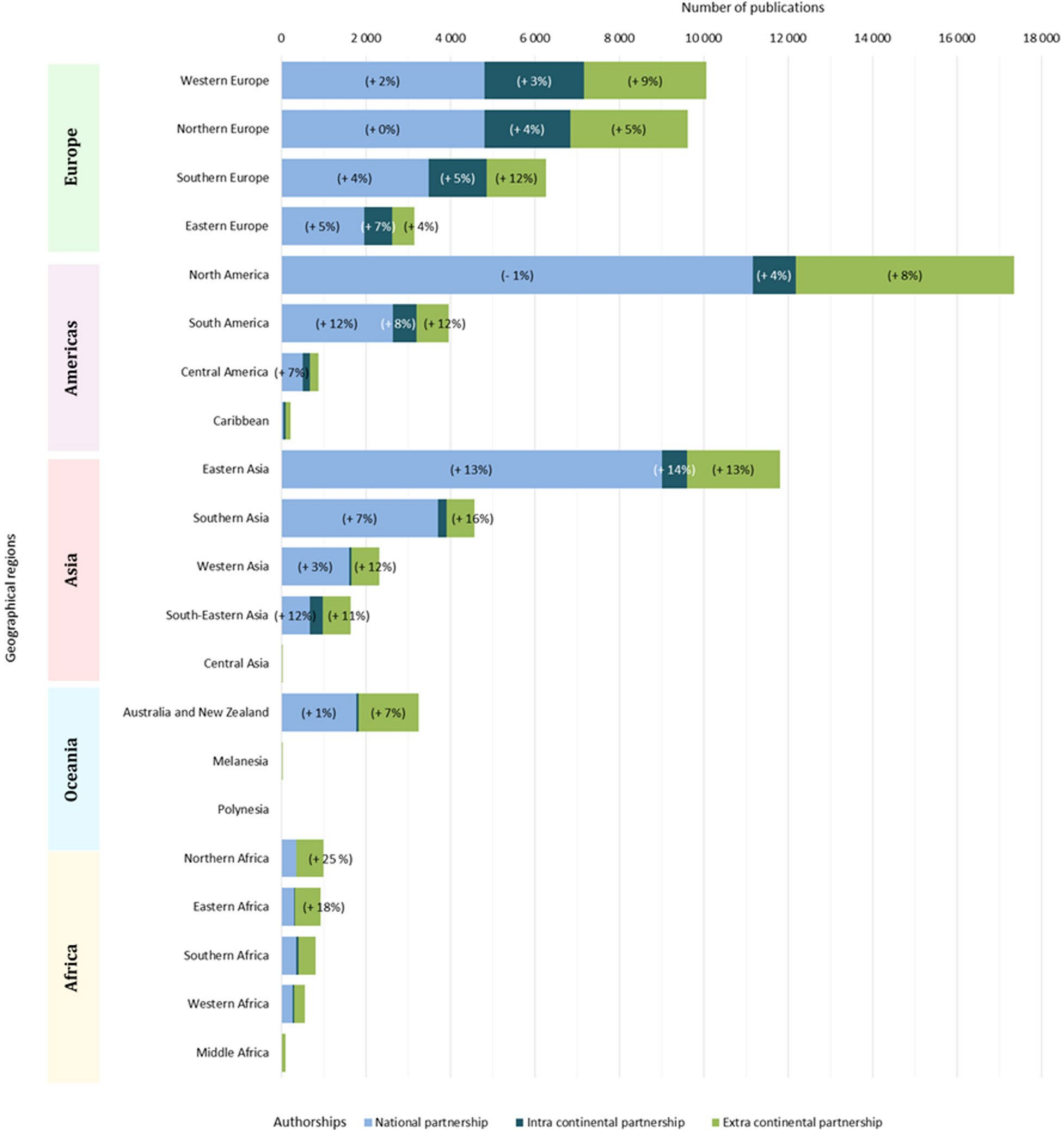
**Distribution of authorship for each continent for the period 2006–2013 (** ***in brackets*** **, significant average annual growth rate, for the period 2006–2013).** To affiliate an article with a continent, at least one author must be from that continent (continent defined by the United Nations). If an article has multiple authors from different continents, it may be affiliated with each of those continents, and thus be counted multiple times.

The distribution of the number of papers in 22 different geographical regions was determined based on the definition established by the United Nations Statistics Division [7] (Figure 10). More details on authorship at the regional level are provided in Figure 11. These proportions varied between regions, but one needs to account for the effect of country size to interpret these figures; by nature, regions with huge countries such as Eastern Asia (with China) or North America (with the United States) have a logical tendency to have a higher percentage of articles with “national partnership” and a lower percentage of articles with “intra-continental partnership” compared to other regions. The average annual growth rate for articles whose authors were from at least two different continents was 9%; the number of such publications per year doubled in 8 years.

#### Co-authorship networks at the regional scale

**Figure 12:**
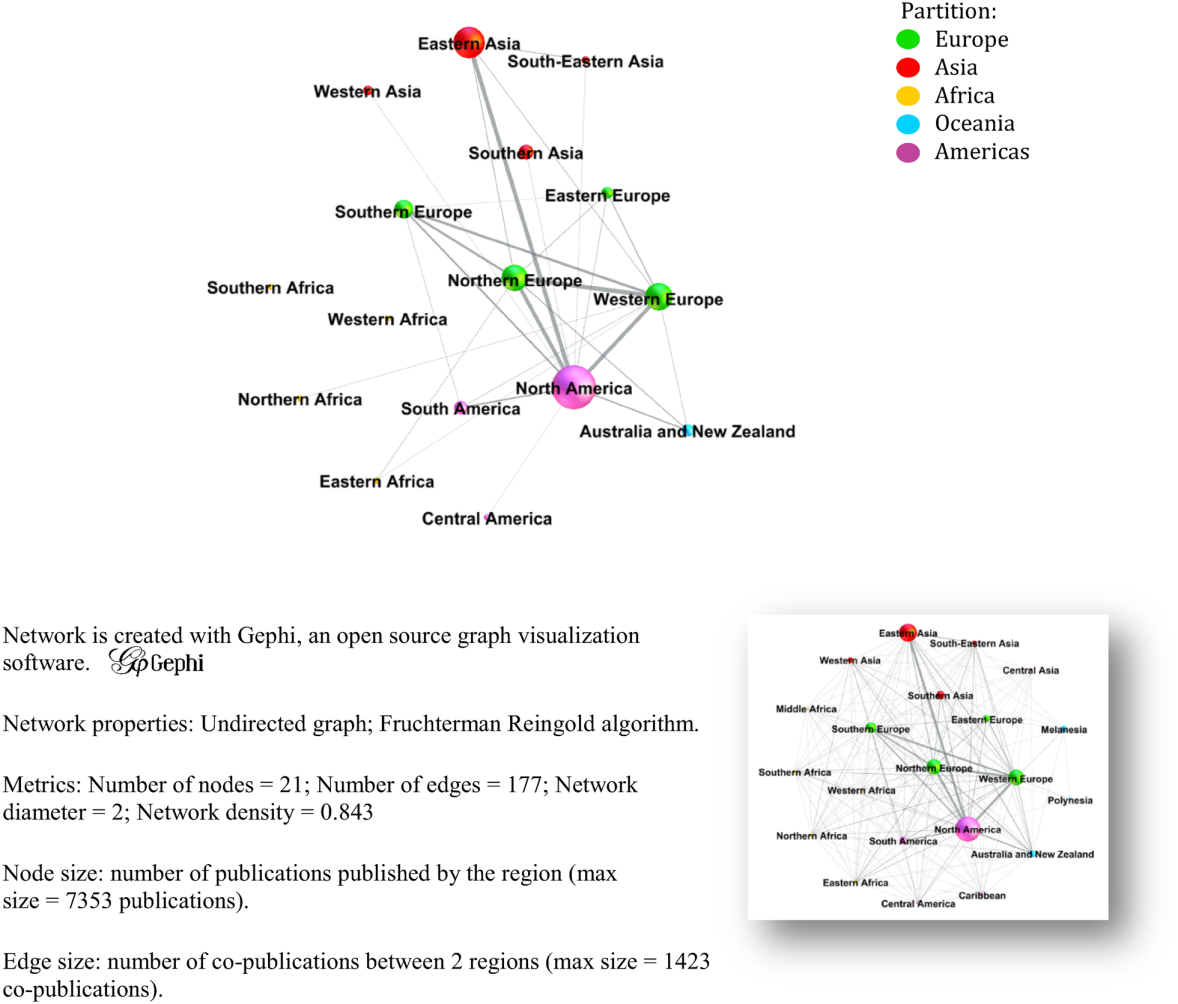
**Co-authorship network of publications between geographical regions for the period 2006–2013** **(Data base** **=** **62** **754 articles; Node threshold** **=** **400 publications; Edge threshold** **=** **200 co-publications).** Geographical regions are defined by the United Nations.

The graph in Figure 12 presents both the volume of scientific articles produced in each geographical region (size of the nodes) and the volume of collaborations (co-authorship) between regions (thickness of the edges). A paper with all of the authors from the same region is counted once in the region of the authors’ origin; it appears in the node of the region. A paper with authors from two different regions is counted twice (once in each of the two regions); it appears in the node of each of the two regions as well as in the edge between the two regions. The entire graph is presented in the small inset below the main graph. In the main figure, only nodes and edges above a defined threshold are presented to highlight the most relevant connexions. The graph shows a well-established network of international collaborations with different levels of volumes and inter-regional collaborations. North America is collaborating with nearly every region, with special emphasis regarding Europe and Asia. European regions largely collaborate with each other as well as with North America. Asia appears to be predominantly connected to North America.

## Discussion

We analyzed the worldwide scientific literature related to infectious diseases of livestock animals. We used the Web of Science, Core collection^®^, an internationally recognized source of data which already has been used by others conducting bibliometric analyses of veterinary research [8, 9]. We chose to consider only papers fully written in English. Indeed, the WOS indexation of journals is considered a criteria of quality. Our purpose was to focus on papers published in international peer-reviewed journals with high publication standards. However, our study does not account for all of the literature published in national journals and written in national langages, which can represent a considerable part of the scientific literature in certain countries, Russia being one example.

### Volume of scientific literature

Currently more than 10 000 papers are published annually worldwide in the field of infectious diseases in livestock animals. However, this amount increases each year, and the average annual growth rate is 5%, which is in the same range as the 4.5% average annual growth rate reported by Pavlech [10] in her recent 12-year survey of the publications of the Journal of Verterinary Internal Medicine. These growth rates for animal health literature are in the same range of magnitude as the overall growth rate recorded in the Web of Science^®^ for all scientific disciplines considered together.

### Research themes

The research themes with an average annual growth rate of less than 5% consequently are considered, in this study, as topics in decline compared to others. Among the themes of research that are increasing faster than the average, we can point to research on viruses. This was observed for all animal species but particularly pig and poultry. Concerning species, we observe an increasing interest in monogastric species (pigs and poultry) as well as fish and honeybees. This is in total accordance with market trend analyses and inter-governmental agencies studies which predict that poultry and pigs will be the leading livestock production sectors in the future [11]. Research efforts seem to already be shifting toward these leading species. We also can highlight some specific domains that are increasing, such as parasitology on poultry, horses and rabbits, and bacteriology on fish.

These increasing fields of research reflect the increasing importance of emerging viral diseases [12] that represent a threat for both animal and human health in a globalized world and in a one health perpective. Another driver is international concern over declining bee populations.

On the other side, research on ruminants and rabbits is declining compared to research on monogastric diseases. Published investigations on prions also are decreasing sharply (−5% per year). This might be related to the fact that as soon as a sufficient research effort has been achieved, thus lowering public health risks, the incentives for such investigations are discontinued, regardless of whether the main scientific questions have or have not been answered.

The observed evolutions will progressively equilibrate the amount of investigations conducted on ruminants compared to those on other animal production species (pigs, poultry, fish). Until now, ruminants represented the major contributors to the literature. We note that the amount of investigations in virology has increased. They might be benefiting from a reorientation of the teams previously working on prions.

### Continent scale

The distribution of the scientific literature among continents reveals three major groups of equivalent contributions, Europe, the Americas and Asia. Africa represents a small 4% of the scientific literature, but it is increasing quite rapidly with a 13% annual growth rate, especially through intercontinental collaborations. Another important point is the contrasting annual increasing rate of the scientific literature between continents; Africa (see above) and Asia (11% annual growth rate) show a much more rapid increase than other continents. Assuming that the current average annual growth rate of each continent will remain steady in coming years, we can make a projection for the situation in 2020. Applying the observed average growth rate per continent to the total number of papers published in 2012 in each continent (last year with complete data), the annual scientific literature would reach almost 13 800 papers in 2020, distributed in a very different way compared to the current situation : 42% in Asia (versus 27% in the 2006–2013 period), 27% in Europe, 21% in the Americas, and the remaining 10% in Africa and Oceania. It will be worthwhile to analyse whether, in the light of the future Asian leadership regarding publications, the overall pattern of topic coverage evolves according to the dietary habits or cultural traits of these main contributors [11].

### International collaborations

At the world level, only 25% of the papers published in the 2006–2013 period showed international co-authorship. However, the average annual growth rate of those papers with inter continental collaborations is higher than average (9%), which will modify the situation in the coming years towards more international co-authorship of the papers. The papers with international co-authorship show a quite diversified network of international relationships, with North America and Europe as the major linking spots. Based on these data, it is very difficult to estimate which part of the international collaborations results from the effort made by various continental and international initiatives organized by funding agencies to promote research collaborations between countries, in comparison with international collaborations initiated by researchers without any incentive.

## Conclusions

The trends observed predict a rapid shift in the geographical poles of importance in research on infectious diseases in livestock animals, with Asia producing almost half of the scientific literature 10 years from now; this will be accompanied by a shift in the species of interest to more research on poultry and pigs. Furthermore, the evolution of the topics of interest over time show a rapid adaptation of research to emerging subjects. These results should be interpreted in view of a very potent and universal driver which strongly influences the topic selection process: financial incentives, namely grant proposals, which are made available by research funding agencies. The scientific literature published in a given year is the direct consequence of priority funding given 8–10 years previously. As an illustration, the 13% annual growth rate of the literature dealing with influenza in the 2006–2013 period is the direct consequence of the efforts made to promote such studies following several influenza outbreaks, including the H5N1 and H1N1 epidemics. Additionally, fad effects may influence topic selection by researchers. A side effect is that pathogen agents and diseases that have fallen out of fashion are no longer considered, resulting in a loss of skills that can become dangerous. This study shows that the conduct of research tends to be driven by short term considerations. The results of this bibliometric analysis could be of interest for future gap analyses on research topics in order to prioritize further research needs.
